# Optimal first-line chemotherapeutic treatment in patients with locally advanced or metastatic esophagogastric carcinoma: triplet *versus* doublet chemotherapy: a systematic literature review and meta-analysis

**DOI:** 10.1007/s10555-015-9576-y

**Published:** 2015-08-13

**Authors:** N. Haj Mohammad, E. ter Veer, L. Ngai, R. Mali, M. G. H. van Oijen, H. W. M. van Laarhoven

**Affiliations:** Department of Medical Oncology, Academic Medical Center, F4-222 Meibergdreef 9, PO box 22600, 1100 DD Amsterdam, The Netherlands

**Keywords:** First-line treatment, Triplet chemotherapy, Doublet chemotherapy, Palliative chemotherapy, Esophageal cancer, Gastric cancer

## Abstract

There is a debate whether triplet or doublet chemotherapy should be used as a first-line treatment in patients with advanced or metastatic esophagogastric cancer. Therefore, here we will review the available literature to assess the efficacy and safety of triplet *versus* doublet chemotherapy as a first-line treatment in patients with advanced esophagogastric cancer. We searched MEDLINE, Embase, and CENTRAL (Cochrane Central Register of Controlled Trials) between 1980 and March 2015 for randomized controlled phase II and III trials comparing triplet with doublet chemotherapy and abstracts of major oncology meetings from 1990 to 2014. Twenty-one studies with a total of 3475 participants were included in the meta-analysis for overall survival. An improvement in overall survival (OS) (hazard ratio (HR) 0.90, 95 % confidence interval (CI) 0.83–0.97) and progression-free survival (PFS) (HR 0.80, 95 % CI 0.69–0.93) was observed in favor of triplet. In addition, the use of triplet was associated with better objective response rate (ORR) (risk ratio 1.25, 95 % CI 1.09–1.44) compared to doublet. The risks of grade 3–4 thrombocytopenia (6.2 vs 3.8 %), infection (10.2 vs 6.4 %), and mucositis (9.7 vs 4.7 %) were statistically significantly increased with triplet compared to doublet. This review shows that first-line triplet therapy is superior to doublet therapy in patients with advanced esophagogastric cancer. However, the survival benefit is limited and the risks of grade 3–4 thrombocytopenia, infection, and mucositis are increased.

**Electronic supplementary material:**

The online version of this article (doi:10.1007/s10555-015-9576-y) contains supplementary material, which is available to authorized users.

## Introduction

Gastric and esophageal cancers are respectively the second and the sixth most common cause of cancer-related deaths worldwide. The only potentially curative option involves resection. Unfortunately, the majority of patients presents with advanced disease or develops metastases after treatment with curative intent. In these patients, palliative systemic chemotherapy improves survival and quality of life, compared to best supportive care [1–3].

Combination therapies have been associated with substantially higher response rates and survival compared to monotherapy [4, 5]. However, the optimal regimen for first-line palliative chemotherapy has yet to be clearly established and the question whether a three-drug regimen is more effective than a potentially less toxic doublet is a point of debate. A Cochrane review published in 2010 concluded that “two and three-drug regimens including 5-FU, cisplatin, with or without an anthracycline are reasonable treatment options [6].” This ambiguity is reflected in various guidelines. According to the National Comprehensive Cancer Network (NCCN) guidelines of 2015 two-drug regimens are preferred and three-drug cytotoxic regimens should be reserved for medically fit patients with good performance scores and access to frequent toxicity evaluation [7]. The European Society for Medical Oncology (ESMO) guidelines of 2013 state that “combination regimens incorporating a platinum agent and a fluoropyrimidine are generally used. It remains controversial whether a triplet regimen is needed [8].” In recently published randomized trials introducing targeted therapies in first-line treatment, mainly doublets have been used as the backbone chemotherapy [9–11], although one trial used a triplet [12].

Therefore, here we will systematically review the existing literature on triplet or doublet therapy in terms of overall survival, progression-free survival, objective response rate, and safety in the management of advanced esophagogastric cancer.

## Methods

### Search methods

A search was conducted at the Cochrane Central Register of Controlled Trials (CENTRAL), MEDLINE, and EMBASE up to March 2015. The search strategy contained medical subject headings (MESH) and text words for esophageal and gastric cancer and all established chemotherapy compounds in esophageal and gastric cancer. We searched all abstracts from the American Society of Clinical Oncology (ASCO) and the ESMO conferences held between 1990 and 2014. The research question was registered in PROSPERO in September 2014 (registration: CRD42014014480).

### Study selection

Randomized phased II or III studies were included. We included studies in abstract form only if information on study design, characteristics of participants, interventions, and outcomes was available in English. We excluded crossover studies and quasi randomized studies. Patients had advanced, recurrent, or metastatic adenocarcinoma of the distal esophagus, gastro-esophageal junction, or stomach. They were not previously treated with chemotherapy (or ≥6 months ago in adjuvant setting). Treatment was defined as intravenous or oral chemotherapy and we excluded targeted therapy/biological therapy. Subgroups were made and named after the third compound that was added to the identical backbone in both arms. One subgroup “other” was created that contained a triplet and a doublet without the presence of an identical doublet backbone.

### Data extraction

NHM, MA, and EV conducted the search. NHM and EV independently scrutinized titles and abstracts and if applicable the full articles. HvL decided in case of disagreement between NHM and EV. NHM, RM, and EV extracted the study characteristics and outcome data. The primary outcome was overall survival (OS). Overall survival was defined as the time between date of randomization and date of death or last date of follow-up.

Secondary outcomes were progression-free survival (PFS), objective response rate (ORR), and toxicity. Treatment-related toxicity was defined as the highest grade of toxicity per participant. Toxicity data, when available, were recorded if scored as grade 3–4 toxicity.

### Assessment of risk of bias

All selected studies were critically appraised using an assessment form designed for the topic of this review according to the *Cochrane Handbook for Systematic Reviews of Interventions* 4.2.2 [13]. Risk of bias caused by the absence of blinded review of CT scans was not scored as high risk, since our primary outcome OS would not be influenced by this parameter. NHM and EV assessed the risk of bias. HvL decided in case of disagreement. If data were missing, we contacted the first author to obtain further information.

### Statistical analysis

Survival analysis was conducted using the intention-to-treat population. A fixed model was used to calculate the pooled hazard ratio (HR) estimate. HRs for mortality were combined using an inverse variance method based on a logarithmic conversion; 95 % confidence intervals were used to determine the standard error using according to Tierney *et al.* [14]. The traditional *Q* test and the *I*^2^ statistic were used to evaluate heterogeneity [15]. Where heterogeneity levels were moderate or high (defined as *I*^2^ ≥ 50 %), we used a random effects model. We repeated the primary analysis and investigated the influence of risk of bias, continent of conduction of the trial, and studies that compared triplet *versus* doublet without identical backbones, excluding those trials with a high risk of bias score, trials conducted in Asia, and trials of which the backbone was not identical. All meta-analyses were performed with Cochrane Review Manager, version 5.3.

## Study outcomes

### Description of studies

**Fig. 1:**
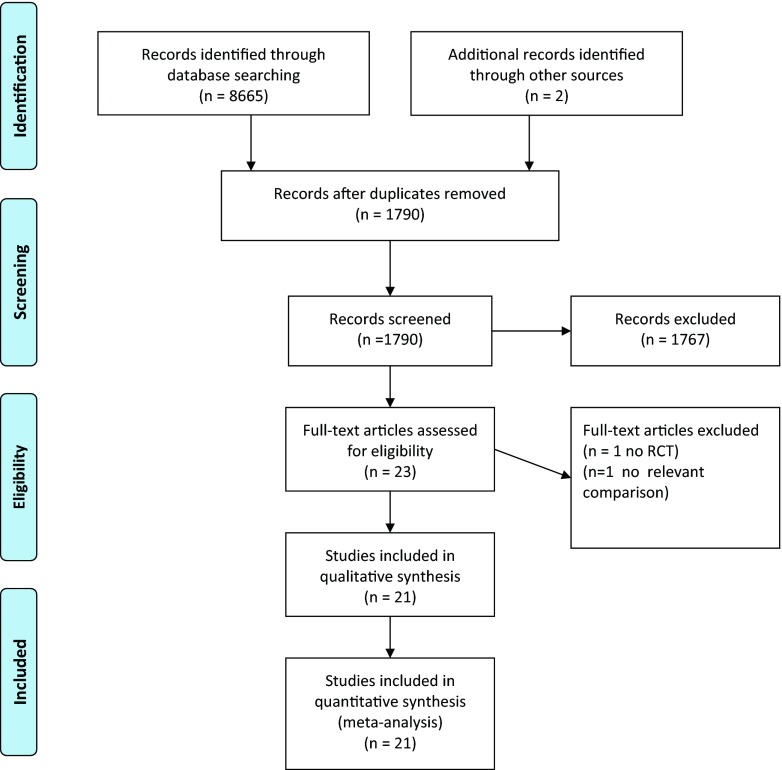
PRISMA flow diagram of literature search and study selection

We identified 6715 articles from the database search. After duplication, 1490 articles were screened on title and abstract. Of these papers, 1467 were excluded: no randomized controlled trials, reviews, and no comparison of a doublet *versus* a triplet. Twenty-three articles were scrutinized as full text. Finally, 21 randomized controlled studies were included in the qualitative and quantitative analysis (Fig. 1.)

Twenty-two studies with in total 3475 participants investigating a triplet *versus* a doublet were included. Table 1 shows the characteristics of the studies included in the meta- analysis.

**Table 1:** Characteristics of the included studies

Study	Number	Arms	Efficacy		Age		Sex		Disease status				ECOG			
			OS	PFS	Median	Range	Male		LA		ME		0–1		≥2	
			Median months				*N*	%	*N*	%	*N*	%	*N*	%	*N*	%
Ajani 2005 [16]	79	DTX + Cis + FU	9.6	NA	57	21–83	53	70	4	6	72	95	79*	100	0	0
	76	DTX + Cis	10.5	NA	57	30–76	61	77	1	1	75	95	75	99	1	1
Al-Batran 2013 [17]	72	DTX + Ox + FU + LV	17.3	9.1	69	65–81	51	71	22	31	50	69	67	93	5	7
	71	Ox + FU + LV	14.4	6.7	70	65–82	45	63	22	32	49	68	65	92	6	9
Cullinan1985 [35]	51	FU + Doxo + MMC	NA	NA	60^#^	NA	39	76	20	39	31	61	32	63	19	37
	49	Doxo + FU	NA	NA	63^#^	NA	37	76	18	37	31	63	33	67	16	33
Douglass 1984 [36]	39	FU + Doxo + Me	24.5	NA	59.5	43–76	28	71	0	0	39	100	30	77	9	23
	46	FU + Doxo + MMC	29.5	NA	61.0	32–81	35	76	0	0	46	100	30	65	16	35
	48	FU + Me	13.5	NA	62.0	24–79	38	80	0	0	48	100	35	72	13	28
	46	Doxo + MMC	19.0	NA	58.0	33–78	37	80	0	0	46	100	28	61	18	39
Guimbaud 2014 [18]	209	Epi + Cis + Cape	9.5	5.3	61	28–84	154	74	36	17	173	83	169	81	36	17
	207	FU + Iri	9.7	5.7	61	29–81	155	75	31	15	176	85	173	84	27	13
Kim 1993 [37]	110	FU + Doxo + MMC	6.84	NA	54	19–77	68	62	NA	NA	NA	NA	75	68	23	21
	112	Cis + FU	8.61	NA	51	20–68	71	63	NA	NA	NA	NA	83	74	20	18
Kim 2001 [38]	60	Epi + Cis + FU	8.5	NA	55	NA	45	75	3	5	57	95	54	90	6	10
	60	Cis + FU	7.3	NA	56	NA	42	70	3	5	57	95	53	88	7	12
Koizumi 2004 [39]	33	5-DFUR + Cis + MMC	8.03	NA	58	36–79	19	58	NA	NA	NA	NA	16	48	13	39
	29	5-DFUR + Cis	5.97	NA	58	37–79	17	59	NA	NA	NA	NA	25	86	6	24
KRCCG 1992 [40]	25	Epi + Cis + FU	6.9	NA	NA	NA	NA	NA	NA	NA	NA	NA	NA	NA	NA	NA
	22	Cis + FU	4.0	NA	NA	NA	NA	NA	NA	NA	NA	NA	NA	NA	NA	NA
Li 2011 [41]	50	PTX + Cis + FU	10.6	NA	59	20–74	32	68	22	46	28	56	NA	NA	NA	NA
	44	Ox + FU	9.9	NA	58	20–75	31	70	17	41	27	61	NA	NA	NA	NA
Lin 2009 [42]	13	FU + Ox + PTX	NA	NA	55	36–67	18	72	NA	NA	NA	NA	NA	NA	NA	NA
	12	FU + Iri	NA	NA	55	36–67	18	72	NA	NA	NA	NA	NA	NA	NA	NA
Maiello 2011 [43]	36	Epi + Cis + Cap	NA	NA	58	39–74	22	60	NA	NA	NA	NA	NA	NA	NA	NA
	31	DTX + FU	NA	NA	61	44–75	23	74	NA	NA	NA	NA	NA	NA	NA	NA
Park 2008 [19]	45	Cis + Iri + FU	10.5	6.2	52	29–70	30	67	0	0	45	100	38	84	7	16
	46	Iri + FU	10.7	4.8	55	26–73	30	67	0	0	45	100	35	78	11	29
Roth 1999 [44]	61	Epi + Cis + FU	9.6	NA	54	NA	37	61	12	22	42	78	24	39	30	61
	61	Epi + FU	7.1	NA	56	NA	42	69	16	30	40	84	27	44	29	56
Roth 2007 [20]	40	Epi + Cis + FU	8.3	NA	59	32–71	30	75	7	17	33	83	40	100	0	0
	38	DTX + Cis	11.0	NA	58	40–70	29	76	7	18	31	82	38	100	0	0
	41	DTX + Cis + FU	10.4	NA	61	35–78	30	73	2	5	39	95	41	100	0	0
Thuss-Patience 2005 [45]	45	Epi + Cis + FU	9.7	NA	63	33–75	36	80	1	2	44	98	44	98	1	2
	45	DTX + FU	9.5	NA	62	34–75	29	64	1	2	44	98	42	95	2	4
Van Cutsem 2006 [22]	227	DTX + Cis + FU	9.2	NA	55	26–79	159	72	6	3	213	96	218	99	3	1
	230	Cis + FU	8.6	NA	55	25–76	158	71	6	3	217	97	221	99	3	1
Van Cutsem 2015 [21]	89	DTX + Ox + FU	14.6	7.6	58	NA	61	69	0	0	89	100	87	98	2	2
	86	DTX + Ox + Cap	11.3	5.6	59	NA	64	74	0	0	86	100	83	97	3	3
	79	DTX + Ox	9.0	4.5	59	NA	51	65	0	0	79	100	77	99	1	1
Van Hoefer 2000 [23]	133	FU + Doxo + MTX	6.7	3.3	58	30–74	96	72	22	17	111	83	117	88	16	12
	134	Cis + FU	7.2	4.1	57	24–74	91	68	21	16	113	84	114	85	20	15
	132	Eto + FU + LV	7.2	3.3	59	25–74	90	68	22	17	110	83	120	91	12	9
Wang 2015 [46]	119	DTX + Cis + FU	10.2	7.2	57	19–80	81	68	30	25	89	75	115	97	4	3
	115	Cis + FU	8.5	4.9	56	33–74	88	77	26	23	89	77	108	94	7	6
Yun 2010 [47]	44	Epi + Cis + Cap	13.8	6.5	55	35–71	28	64	NA	NA	NA	NA	40	91	1	9
	47	Cis + Cap	12.7	6.4	58	33–75	34	72	NA	NA	NA	NA	41	87	4	13

*OS* overall survival, *PFS* progression-free survival, *TTP* time to progression, *LA* locally advanced, *ME* metastatic disease, *ECOG* Eastern Cooperative Oncology Group performance status, *NA* not applicable, *DTX* docetaxel, *PTX* palictaxel, *Cis* cisplatin, *FU* fluorouracil, *Cap* capecitabine, *5-DFUR* doxifluridine, *Ox* oxaliplatin, *Doxo* doxorubicin, *Epi* epirubicin, *Iri* irinotecan, *MTX* methotrexate, *MMC* mitomycin C, *Eto* etoposide

*Karnofksi 80-100

# mean age OR

### Overall survival, progression-free survival, and objective response rate

**Fig. 2:**
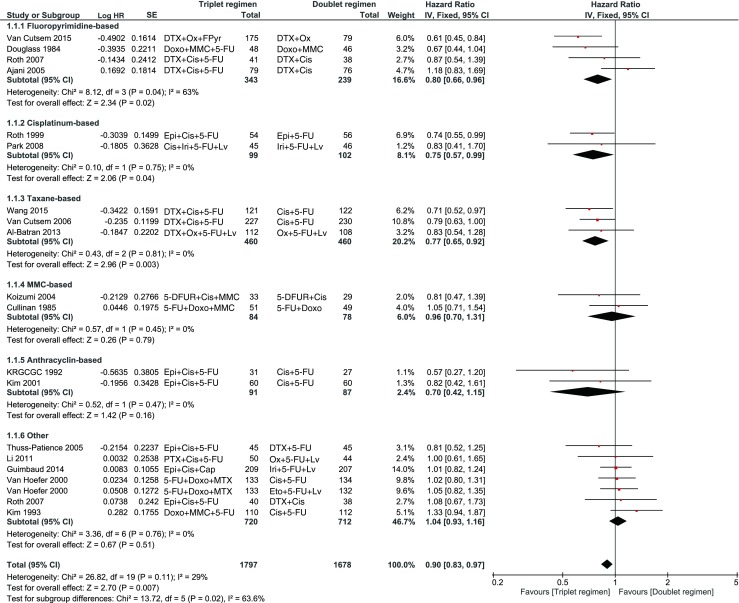
When examining the subgroups, taxane and cisplatin showed a significant benefit

**Fig. 3:**
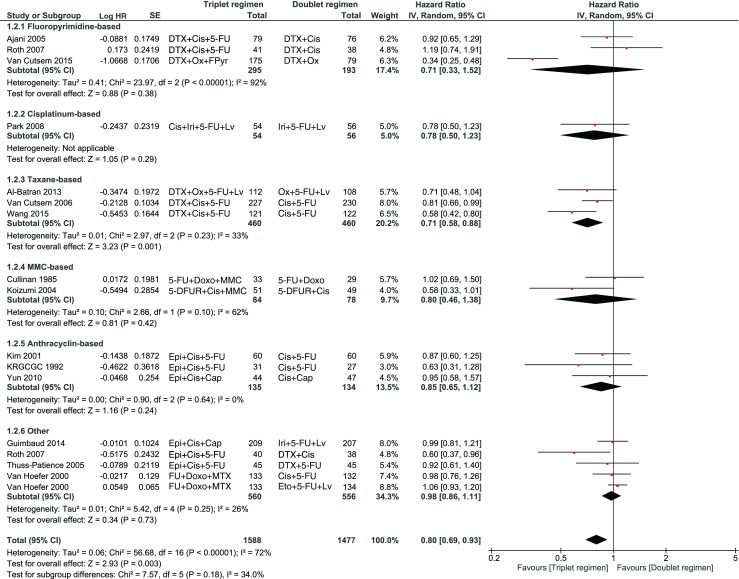
A significant benefit was observed for PFS in favor of a triplet, which was mainly based on the addition of a taxane to the doublet

A significant improvement in OS with a low heterogeneity was observed in favor of a triplet (HR 0.90, 95 % confidence interval (CI) 0.83–0.97, *I*^2^ = 29 %). When examining the subgroups, especially the triplets with fluoropyrimidine, taxane and cisplatin showed a significant benefit (Fig. 2.). Also, a significant benefit was observed for PFS in favor of a triplet (HR 0.80, 95 % CI 0.69–0.93; Fig. 3), which was mainly based on the addition of a taxane to the doublet.

**Fig. 4:**
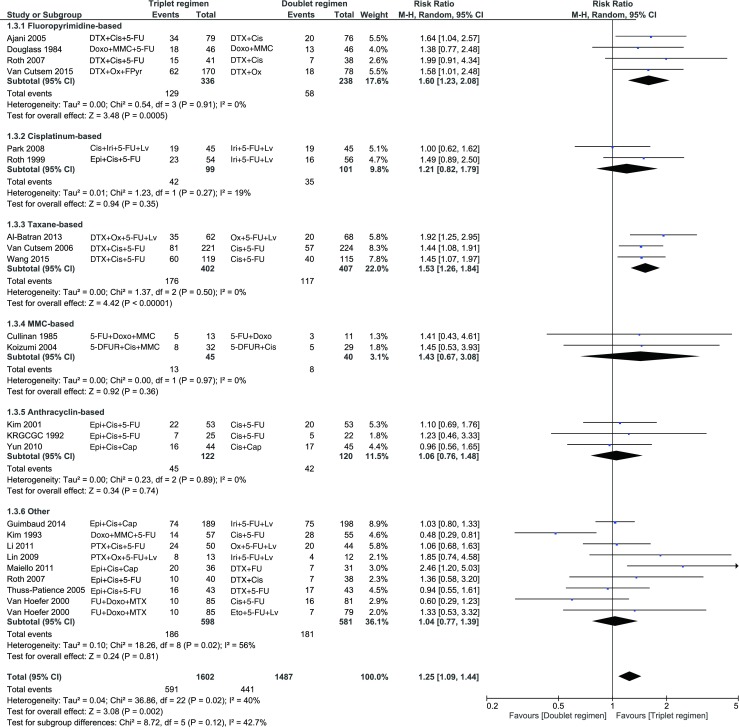
The use of a triplet was associated with a better ORR compared to a doublet, which was mainly due to triplets with a fluoropyrimidine or taxane

In addition, the use of a triplet was associated with a better ORR compared to a doublet (risk ratio = 1.25, 95 % CI 1.09-1.44). This was mainly due to triplets with a fluoropyrimidine or taxane (Fig. 4.)

### Risk of bias and sensitivity analyses

**Fig. 5:**
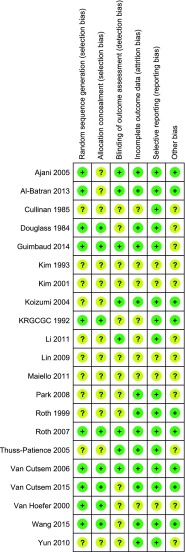
Risk of bias assessment

Data on outcome measures and risk of bias were requested from all authors, but unfortunately only 28 % of the corresponding authors provided further information. Risk of bias assessments are shown in supplementary table 1 (Fig. 5).

**Fig. 6:**
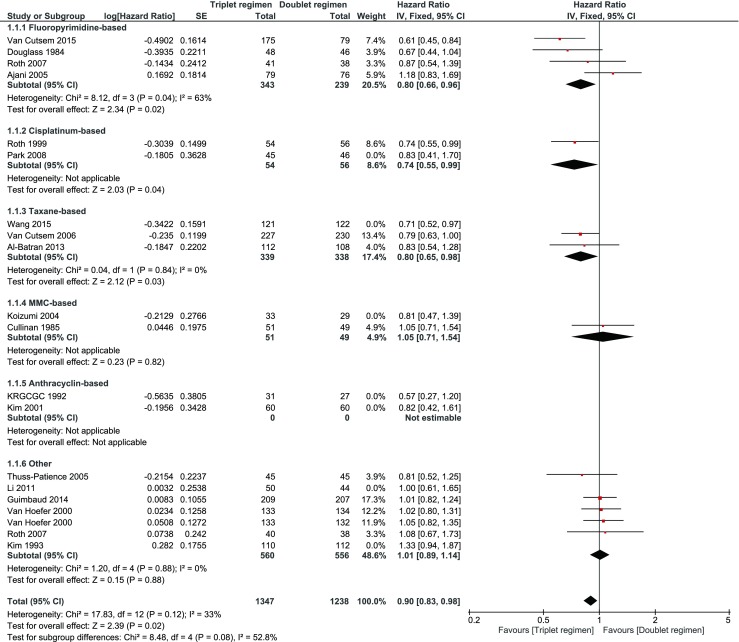
Sensitivity analysis excluding those trials that were conducted in Asia

**Fig. 7:**
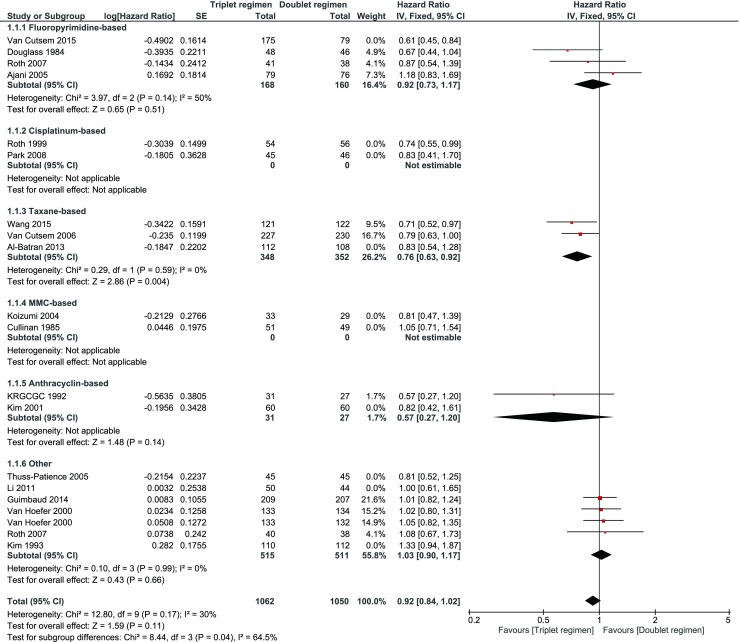
Sensitivity analysis excluding studies with “unknown” risk of bias on “random sequence” and “allocation concealment”

**Fig. 8:**
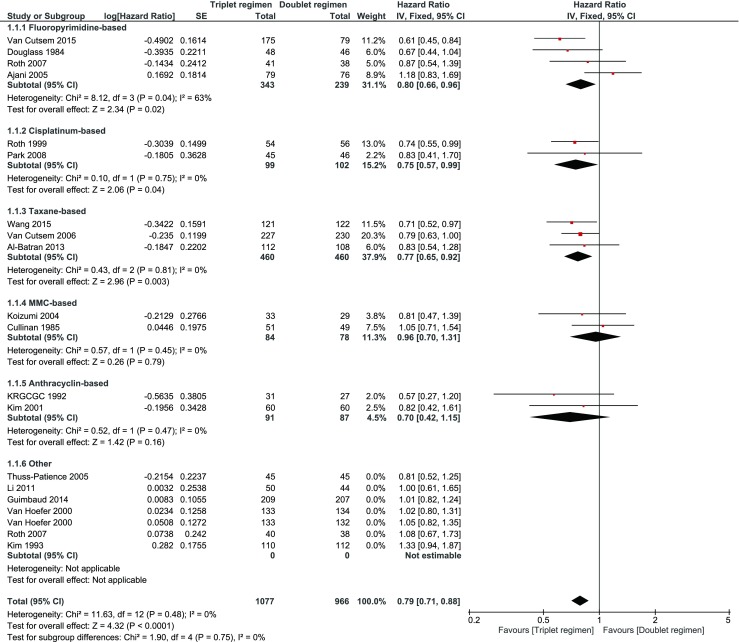
Sensitivity analysis excluding those trials that compared a triplet *versus* a doublet without the presence of two identical compounds in both arms

A sensitivity analysis excluding those trials that were conducted in Asia had no impact on the HR (0.90, 95 % CI 0.93–0.97) for OS (Fig. 6). Sensitivity analysis excluding trials on the basis of low quality was not possible due to the lack of data. Instead, studies with “unknown” risk of bias on “random sequence” and “allocation concealment” were excluded in a sensitivity analysis that showed a comparable HR of 0.92 (95 % CI 0.84–1.02) (Fig. 7). Sensitivity analysis excluding those trials that compared a triplet *versus* a doublet without the presence of two identical compounds in both arms showed a lower HR for OS of 0.79 (95 % CI 0.71–0.88 (Fig. 8)).

### Toxicity

The risk of grade 3–4 thrombocytopenia (6.2 vs 3.7 %), infection (10.2 vs 6.4 %), and mucositis (9.7 vs 4.7 %) was significantly increased with a triplet compared to a doublet (Table 2).

**Table 2:** Toxicity grade 3 or 4

Toxicity grade 3 or 4	Triplet				Doublet			
	*N*	Total	%	*N*	Total	%	RR	95 % CI
Hematologic toxicity								
Anemia	106	840	12.6	121	823	14.7	0.86	0.68–1.09
Neutropenia	543	1006	54.0	470	986	47.7	1.07	0.92–1.23
Neutropenic fever	46	385	11.9	46	367	12.5	0.95	0.50–1.82
Thrombocytopenia	61	986	6.2	37	962	3.8	1.57^a^	1.06–2.31
Non-hematologic toxicity								
Fatigue	50	331	15.1	52	316	16.5	0.91	0.64–1.29
Infection	64	630	10.2	40	629	6.4	1.60^a^	1.09–2.33
Mucositis	59	607	9.7	28	591	4.7	2.20^a^	1.00–4.86
Nausea	85	665	12.8	63	648	9.7	1.34	0.98–1.82
Vomiting	84	728	11.5	81	716	11.3	1.04	0.78–1.38
Diarrhea	114	1260	9.0	98	1244	7.9	0.98	0.60–1.61
Toxicity-related deaths	68	1069	6.4	54	1052	5.1	1.24	0.89–1.74

*RR* relative risk, *95 % CI* 95 % confidence interval

^a^Significant

## Discussion

Our systematic review and meta-analysis showed that a triplet regimen was superior to a doublet regimen in terms of overall survival, progression-free survival, and objective response rate. However, hazard ratios were of limited clinical relevance and toxicity grades 3 and 4 were significantly higher in the triplet regimens.

The positive effects on OS of adding a fluoropyrimidine or a taxane to a doublet were based on a relatively large number of patients (>500 patients), making the findings in these subgroups very robust. The favorable outcome of a triplet with cisplatin over a doublet was based on a smaller number of patients. Our results are in line with the previously published findings by Wagner *et al.* [6] and are considered clinically relevant given the widespread use of fluoropyrimidine, taxane, and cisplatin containing triplets in routine practice in Europe [16–23].

This contrasts with our finding that the addition of an anthracycline to a doublet did not meet statistical significance for OS. Although the addition of anthracycline to the doublet showed an HR of 0.7, this was not significant and the number of included patients did not reach 200 in total. It should be noted that Wagner *et al.* reported a significant HR of 0.77 (95 % CI 0.62–0.95) for the addition of an anthracycline. This was based on three randomized trials, of which the largest study (81.7 % weight) investigated the comparison of an anthracycline-based triplet with a non-anthracycline-based triplet and showed a significant benefit for the anthracycline-based triplet. The other two included studies were small and compared a triplet *versus* a doublet but did not show significance. Based on Wagner’s data, it may be concluded that an anthracycline-based triplet is superior to a non-anthracycline-based triplet, but based on our results, the true relevance of the addition of anthracyclines to a doublet is doubtful.

In previous meta-analyses, results of PFS and ORR have not been reported. We showed a significant improvement of the secondary endpoint PFS and ORR in favor of triplet chemotherapy, more specifically in the triplets adding a taxane and a taxane or fluoropyrimidine to the combination, respectively. Although in general in metastatic disease ORR is not considered to be the most robust outcome measure, in advanced esophagogastric cancer, ORR may be a clinically relevant end point, given the high symptom burden that patients may suffer from that may be alleviated by response to treatment [1].

Substantial regional differences have been reported between Asian and non-Asian populations in presentation and subtypes of esophagogastric cancer. For example, in several Asian countries, patients present with early disease, whereas in Western countries the majority of patients present with advanced or metastatic disease [24]. Secondly, while intestinal type of gastric cancer is more prevalent in Asian countries, diffuse gastric cancer is more common in non-Asian countries [25]. Nevertheless, a sensitivity analysis excluding the trials conducted in Asia did not change our results. The sensitivity analysis on the basis of quality of studies in which we excluded the studies with unknown risk of bias may be very firm and can give an underestimation of the quality of the excluded studies. As the findings are consistent with those from the primary analysis, the credibility of our results is strengthened.

Finally, the sensitivity analysis excluding the trials that did not compare at least two identical compounds in both arms did not cause a relevant change of the resulting HR with reduced heterogeneity compared to the original analysis. Hence, the results of this comparison can be considered to be highly robust.

In general, toxicity was significantly higher in the triplet combinations compared to the doublet. In the context of palliative treatment, the degree of toxicity is an important aspect that should be taken into account. Hematologic toxicity has been shown to result in substantial psychological and economic burden [26]. However, others have observed that in patients with advanced esophagogastric cancer, toxicity did not directly affect quality of life [1]. Similarly, in a review in colorectal cancer, no correlation was noted between toxicity and quality of life [27].

Although the survival of patients treated with a triplet significantly outweighed the survival of patients treated with a doublet, overall, the survival gain was modest with a hazard ratio of 0.90 of which the clinical relevance may be questioned [28]. However, the benefits of specific triplets, such as fluoropyrimidine, cisplatin, or taxane-based triplets, were more convincing. It should be noted that in this analysis, the effect of second-line treatment has not been taken into consideration. Since second-line treatment has become an accepted standard of care [29–32], adding a third compound in the first line may have become less relevant and could be reserved for second-line or even third-line treatment. Indeed, sequential chemotherapy has been shown equally effective compared to combination-therapy patients with advanced colorectal cancer [33]. However, in contrast to colorectal cancer, in advanced esophagogastric cancer, after progression a substantial part of patients may not be sufficiently fit to undergo second-line treatment [4, 9, 34]. This underscores the relevance of clinical studies incorporating new cytotoxic agents into a triplet, which may be less toxic than currently used agents. For example, the currently recruiting ACTION trial assesses the feasibility of adding the new taxane nab-paclitaxel to the combination of capecitabine and oxaliplatin (clinicaltrials.gov NCT02273713).

Several strengths and limitations of this meta-analysis should be acknowledged. First, the findings regarding OS were very robust, demonstrated by the series of sensitivity analyses. Second, we defined subgroups on the basis of the third chemotherapy compound in order to examine the effect of the adding of a specific compound. However, caution is needed, when interpreting the beneficial effect of triplet *versus* doublet in terms of PFS because PFS was a secondary outcome in only a limited amount of studies. Moreover, the majority of included studies were phase II studies. Missing information regarding the PFS may under- or overestimate the gain in PFS. Second, toxicity was not uniformly scored which precluded extensive analysis. Third, quality of life was not a designated secondary outcome in most studies, and consequently, no recommendations could be made in this respect. Fourth, not all relevant sensitivity analyses could be conducted as planned, as only 28 % of the authors provided information to evaluate the risk of bias. Fifth, our meta-analysis is not based on individual patient data. Therefore, differences in individual baseline characteristics cannot be adjusted for.

In conclusion, addition of a fluoropyrimidine, cisplatin, or taxane to a doublet showed superior overall survival in first-line treatment of advanced esophagogastric cancer, at the cost, however, of higher toxicity. There is a need for new triplets with cytotoxic agents, which may be less toxic than the currently used regimens.

## Electronic supplementary material

ESM 1 Search strategy. a. search strategy Central, b. search strategy PubMed, and c. search strategy Embase (DOCX 94.6 kb)

ESM 2 Risk of bias assessments (PDF 440 kb)
