# Supplementary material for: Optimal first-line chemotherapeutic treatment in patients with locally advanced or metastatic esophagogastric carcinoma: triplet versus doublet chemotherapy: a systematic literature review and meta-analysis
Source: Cancer Metastasis Rev. 2015 Aug 13;34(3):429–41. doi: 10.1007/s10555-015-9576-y (PMC4573655; doi:10.1007/s10555-015-9576-y)
Supplement: Supplementary file 1 — Search strategy. a. search strategy Central, b. search strategy PubMed, and c. search strategy Embase (DOCX 94.6 kb) [file 10555_2015_9576_MOESM1_ESM.docx]

**Search**: Doublet versus triple therapie bij irresectabel oesofagusmaagcarcinoom

**Database**: Cochrane Central Register of Controlled Trials (CENTRAL) Issue 3

| ID | Search | Hits |
| --- | --- | --- |
| #1 | MeSH descriptor: [Esophageal Neoplasms] explode all trees | 823 |
| #2 | MeSH descriptor: [Stomach Neoplasms] explode all trees | 1372 |
| #3 | ((esophag* or oesophag* or stomach or gastric or gastroesophag* or gastrooesophag*) and (neoplas* or cancer* or carcino* or adenocarcino* or tumor or tumors or tumour or tumours or malig*)):ti,ab,kw | 4455 |
| #4 | #1 or #2 or #3 | 4455 |
| #5 | MeSH descriptor: [Palliative Care] explode all trees | 1245 |
| #6 | MeSH descriptor: [Neoplasm Metastasis] explode all trees | 3392 |
| #7 | (palliat* or advanced or metasta* or irresect* or unresect* or un-resect* or non-resect* or nonresect* or inopera* or non-opera* or nonopera* or unopera*):ti,ab,kw | 31093 |
| #8 | #5 or #6 or #7 | 31149 |
| #9 | MeSH descriptor: [Drug Therapy, Combination] explode all trees | 34018 |
| #10 | MeSH descriptor: [Drug Combinations] explode all trees | 8739 |
| #11 | MeSH descriptor: [Antineoplastic Agents] explode all trees | 8900 |
| #12 | MeSH descriptor: [Anthracyclines] explode all trees | 3793 |
| #13 | MeSH descriptor: [Leucovorin] explode all trees | 926 |
| #14 | MeSH descriptor: [Organoplatinum Compounds] explode all trees | 1252 |
| #15 | MeSH descriptor: [Oxonic Acid] explode all trees | 43 |
| #16 | MeSH descriptor: [Taxoids] explode all trees | 1799 |
| #17 | (chemotherap* or polytherap* or polychemotherap* or combination* or two-agent* or two-drug* or double-drug* or doublet* or three-agent* or three-drug* or triple* or multi-agent or multi-drug or active agent* or antineoplastic* or anti-neoplastic* or anticancer* or anti-cancer* or antitumor* or anti-tumor* or antitumour* or anti-tumour* or anthracyclin* or capecitabine or carboplatin* or cisplatin* or docetaxel or doxorubicin* or epirubicin* or fluoropyrimidine* or fluorouracil or 5-FU or folinic acid or irinotecan or leucovorin* or mitomycin* or organoplatin* or oteracil or oxaliplatin* or oxonic acid or paclitaxel or platin* or S-1 or taxane* or tegafur or trastuzumab):ti,ab,kw | 109603 |
| #18 | #9 or #10 or #11 or #12 or #13 or #14 or #15 or #16 or #17 | 110338 |
| #19 | #4 and #8 and #18 in Trials | 1142 |

Supplementary table 1a Search strategy CENTRAL

**Search**: Doublet versus triple therapie bij irresectabel oesofagusmaagcarcinoom

**Database**: PubMed

("Esophageal Neoplasms"[Mesh] OR "Stomach Neoplasms"[Mesh] OR ((esophag*[tiab] OR oesophag*[tiab] OR stomach[tiab] OR gastric[tiab] OR gastroesophag*[tiab] OR gastrooesophag*[tiab]) AND (neoplas*[tiab] OR cancer*[tiab] OR carcino*[tiab] OR adenocarcino*[tiab] OR tumor[tiab] OR tumors[tiab] OR tumour[tiab] OR tumours[tiab] OR malig*[tiab]))) AND ("Palliative Care"[Mesh] OR "Neoplasm Metastasis"[Mesh] OR palliat*[tiab] OR advanced[tiab] OR metasta*[tiab] OR irresect*[tiab] OR unresect*[tiab] OR un-resect*[tiab] OR non-resect*[tiab] OR nonresect*[tiab] OR inopera*[tiab] OR non-opera*[tiab] OR nonopera*[tiab] OR unopera*[tiab]) AND ("Drug Therapy, Combination"[Mesh] OR "Drug Combinations"[Mesh] OR "Antineoplastic Agents"[Mesh] OR "Antineoplastic Agents"[Pharmacological Action] OR "Anthracyclines"[Mesh] OR "Leucovorin"[Mesh] OR "Organoplatinum Compounds"[Mesh] OR "Oxonic Acid"[Mesh] OR "Taxoids"[Mesh] OR chemotherap*[tiab] OR polytherap*[tiab] OR polychemotherap*[tiab] OR combination*[tiab] OR two-agent*[tiab] OR two-drug*[tiab] OR double-drug*[tiab] OR doublet*[tiab] OR three-agent*[tiab] OR three-drug*[tiab] OR triple*[tiab] OR multi-agent[tiab] OR multi-drug[tiab] OR active agent*[tiab] OR antineoplastic*[tiab] OR anti-neoplastic*[tiab] OR anticancer*[tiab] OR anti-cancer*[tiab] OR antitumor*[tiab] OR anti-tumor*[tiab] OR antitumour*[tiab] OR anti-tumour*[tiab] OR anthracyclin*[tiab] OR capecitabine[tiab] OR carboplatin*[tiab] OR cisplatin*[tiab] OR docetaxel[tiab] OR doxorubicin*[tiab] OR epirubicin*[tiab] OR fluoropyrimidine*[tiab] OR fluorouracil[tiab] OR 5-FU[tiab] OR folinic acid[tiab] OR irinotecan[tiab] OR leucovorin*[tiab] OR mitomycin*[tiab] OR organoplatin*[tiab] OR oteracil[tiab] OR oxaliplatin*[tiab] OR oxonic acid[tiab] OR paclitaxel[tiab] OR platin*[tiab] OR S-1[tiab] OR taxane*[tiab] OR tegafur[tiab] OR trastuzumab[tiab]) AND (randomized controlled trial[pt] OR controlled clinical trial[pt] OR randomized[tiab] OR randomised[tiab] OR placebo[tiab] OR clinical trials as topic[mesh:noexp] OR randomly[tiab] OR trial[ti]) AND (english[la] OR dutch[la])

Supplementary table 1b Search strategy Pubmed

**Search**: Doublet versus triple therapie bij irresectabel oesofagusmaagcarcinoom

**Database**: Embase (Ovid) 1980 to Present

1. esophagus tumor/ or exp esophagus cancer/
2. stomach tumor/ or exp stomach cancer/
3. ((esophag* or oesophag* or stomach or gastric or gastroesophag* or gastrooesophag*) adj5 (neoplas* or cancer* or carcino* or adenocarcino* or tumor or tumors or tumour or tumours or malig*)).ti,ab.
4. or/1-3
5. exp cancer palliative therapy/
6. exp metastasis/
7. advanced cancer/
8. inoperable cancer/
9. (palliat* or advanced or metasta* or irresect* or unresect* or un-resect* or non-resect* or nonresect* or inopera* or non-opera* or nonopera* or unopera*).ti,ab.
10. or/5-9
11. exp drug combination/
12. exp antineoplastic agent/
13. folinic acid/
14. platinum complex/
15. oteracil/
16. taxane derivative/
17. taxoid/
18. (chemotherap* or polytherap* or polychemotherap* or combination* or two-agent* or two-drug* or double-drug* or doublet* or three-agent* or three-drug* or triple* or multi-agent or multi-drug or active agent* or antineoplastic* or anti-neoplastic* or anticancer* or anti-cancer* or antitumor* or anti-tumor* or antitumour* or anti-tumour* or anthracyclin* or capecitabine or carboplatin* or cisplatin* or docetaxel or doxorubicin* or epirubicin* or fluoropyrimidine* or fluorouracil or 5-FU or folinic acid or irinotecan or leucovorin* or mitomycin* or organoplatin* or oteracil or oxaliplatin* or oxonic acid or paclitaxel or platin* or S-1 or taxane* or tegafur or trastuzumab).ti,ab.
19. or/11-18
20. exp controlled clinical trial/ or randomized.ti,ab. or randomised.ti,ab. or placebo.ti,ab. or randomly.ti,ab. or trial.ti.
21. 4 and 10 and 19 and 20
22. limit 21 to (dutch or english)
23. limit 22 to (conference abstract or conference paper or "conference review" or conference proceeding)
24. 22 not 23

Supplementary table 1c Search strategy Embase
