# Supplementary material for: Optimal first-line chemotherapeutic treatment in patients with locally advanced or metastatic esophagogastric carcinoma: triplet versus doublet chemotherapy: a systematic literature review and meta-analysis
Source: Cancer Metastasis Rev. 2015 Aug 13;34(3):429–41. doi: 10.1007/s10555-015-9576-y (PMC4573655; doi:10.1007/s10555-015-9576-y)
Supplement: Supplementary file 2 — Risk of bias assessments (PDF 440 kb) [file 10555_2015_9576_MOESM2_ESM.pdf]

	Random sequence generation (selection bias)	Allocation concealment (selection bias)	Blinding of outcome assessment (detection bias)	Incomplete outcome data (attrition bias)	Selective reporting (reporting bias)	Other bias
Ajani 2005	+	?	+	+	+	+
Al-Batran 2013	+	?	+	+	+	+
Cullinan 1985	?	?	?	?	+	?
Douglass 1984	+	+	?	+	+	?
Guimbaud 2014	+	+	+	+	+	?
Kim 1993	?	?	?	?	?	?
Kim 2001	?	?	?	?	?	?
Koizumi 2004	?	?	+	+	+	+
KRGCGC 1992	+	+	?	?	+	+
Li 2011	?	?	+	?	+	?
Lin 2009	?	?	?	?	?	?
Maiello 2011	?	?	?	?	?	?
Park 2008	?	?	?	+	+	?
Roth 1999	?	?	?	+	+	+
Roth 2007	+	+	+	+	+	+
Thuss-Patience 2005	?	?	+	+	+	?
Van Cutsem 2006	+	+	+	+	+	+
Van Cutsem 2015	+	+	?	+	+	+
Van Hoefer 2000	+	+	?	?	?	?
Wang 2015	+	+	?	+	+	+
Yun 2010	?	?	?	+	+	?
